# Isolation and Purification of Bacterially Produced Polyhydroxyalkanoates: Mechanisms, Limitations, and Current Advances †

**DOI:** 10.3390/life16020269

**Published:** 2026-02-04

**Authors:** Ľubomíra Jurečková, Daniela Chmelová, Miroslav Ondrejovič, Stanislav Miertuš

**Affiliations:** 1 Department of Biotechnology, Faculty of Natural Sciences, University of Ss. Cyril and Methodius, J. Herdu 2, SK-91701 Trnava, Slovakiadaniela.chmelova@ucm.sk (D.C.); or icarst.miertus@libero.it (S.M.); 2 International Centre for Applied Research and Sustainable Technology n.o., Jamnického 19, SK-84101 Bratislava, Slovakia

**Keywords:** polyhydroxyalkanoates, bacteria, scl-PHA, mcl-PHA, isolation, extraction

## Abstract

Polyhydroxyalkanoates (PHAs) are microbial polyesters that belong to a group of bioplastics with the potential to replace petroleum-derived plastics. Their main drawback is the high production cost, which puts them at a disadvantage compared to conventional plastics. A significant part of these costs arises from the isolation of PHAs from the cellular biomass of producing microorganisms. This review summarizes the main approaches used to recover both scl- and mcl-PHAs from native or dried (lyophilized) biomass, with attention to physical, chemical, and biological methods. Key parameters influencing extraction efficiency, polymer purity, and the final material properties are discussed, including pretreatment steps that often determine the overall outcome. The review also compares traditional halogenated solvent extraction with more environmentally acceptable alternatives and considers how different strategies can be combined to improve recovery. The current literature highlights the need for sustainable and economically acceptable processes that would make large-scale PHA production more feasible.

## 1. Introduction

Polyhydroxyalkanoates (PHAs) are biopolyesters of hydroxycarboxylic acids that various microorganisms accumulate inside their cells as a reserve source of carbon and energy. Beyond their role in cellular metabolism, PHA accumulation has been linked to increased stress resilience, as recent evidence shows that PHA-producing organisms exhibit enhanced tolerance to abiotic stress, including repeated freezing and thawing cycles [1]. PHAs form spherical granules in the cytoplasm, and their number and size depend on the organism and the cultivation conditions [2]. The first identified and the most extensively studied representative is poly(3-hydroxybutyrate) (P3HB), a short-chain-length (scl) PHA composed of monomer units with a short carbon chain. P3HB granules contain roughly 97.7% polyesters and about 1.87% proteins, which play both active and passive roles in granule formation and PHA turnover [3].

PHAs have been shown to have beneficial biological responses in various medical and biotechnological applications [4,5,6]. They are biodegradable and, under appropriate conditions, even compostable [7,8]. Their use in medicine is further supported by their biocompatibility. However, biocompatibility should not be considered as an inherent property of PHAs as polymers, but rather as the result of the interaction between the material and the host organisms in a specific application context. This interaction depends on the particular type of PHAs used, which can differ in chemical structure and physical properties [9]. These properties are determined by fermentation conditions, isolation methods, and subsequent processing steps leading to the final form of the product. These features make them a more acceptable option than many conventional plastics, whose accumulation in the environment has become a global concern. Although PHAs can replace petroleum-based plastics in many applications, their large-scale use is still limited by high production costs. The price of these biopolymers is three to four times higher than that of polyethylene or polypropylene [10,11]. This is mainly due to the cost of raw materials and the high expense of isolating and purifying PHA, since the polymer is stored inside microbial cells.

**Figure 1:**
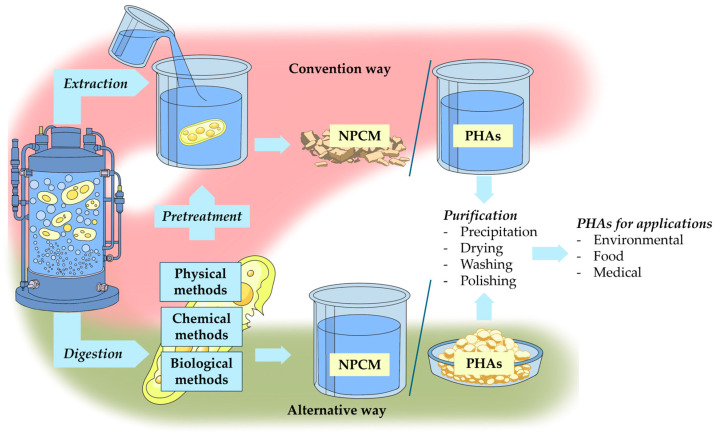
Strategies for PHA isolation from bacterial biomass. NPCM—non-PHA cell mass and PHAs—polyhydroxyalkanoates.

Although using unconventional substrates can reduce production costs to some extent, one of the major challenges in lowering the price of industrial PHA production remains the isolation and purification of the polymer from microbial biomass, which may account for up to 50% of total production costs [12]. Several approaches have been described for recovering PHA from producing cells while preserving the quality of the resulting polymer [13]. The effective isolation of intracellular biopolymers relies on thorough disruption of the cellular structure followed by appropriate purification steps (Figure 1).

The biomass must undergo an isolation process in which either the cell material surrounding the PHA or the polymer itself is solubilized. The PHAs are then separated from the disrupted cell mass and purified according to the requirements of the final product [14]. Separating PHAs from residual biomass containing DNA, RNA, polypeptides, lipids, and peptidoglycans is technically challenging because all of these components remain in the solid phase [15]. Pretreatment and PHA isolation are often inconsistently merged in the literature. In this review, these steps are explicitly separated to enable a more precise comparison of recovery strategies and their impact on extraction efficiency, polymer purity, and PHA properties. The review is based primarily on original experimental studies, preserving methodological detail often lost in the secondary literature. Several approaches with varying efficiency have been described, and they are generally grouped according to their underlying mechanism into physical, chemical, and biological methods [16].

Physical methods include those aimed at removing residual cell biomass, such as milling, high-pressure homogenization (HPH), ultrasonication, or gamma irradiation, as well as methods based on dissolving the target polymer in an appropriate solvent followed by precipitation of the PHA solution. Chemical and biological methods can be collectively referred to as digestion methods, since, unlike solvent extraction, they do not solubilize PHA but instead break down the residual cell material [17]. Based on the catalyst involved, digestion methods are divided into biological digestion, which employs enzymes or whole organisms, and chemical digestion, driven by chemical reagents. Each method has its own advantages and limitations. These can also be combined, which may improve overall performance by taking advantage of the partial contributions of each approach. However, several studies have shown that an inappropriate sequence of methodological steps in a PHA isolation workflow can lead to the conversion of amorphous PHA into its crystalline form. This occurs through the formation of heterogeneous nucleation sites caused by damage to the protective layer on the surface of PHA granules [18,19,20]. The most common reason for this issue is the use of an unsuitable pretreatment method, which can reduce the effectiveness of otherwise well-established isolation procedures, even when the main isolation conditions are properly optimized. It is therefore essential to choose an appropriate isolation method that considers both the producing organism and the specific type of biopolymer being recovered.

The intended application of PHAs (environmental, packaging, or medical uses) determines the required polymer quality in terms of molecular weight, monomer composition, and the acceptable levels of residual components originating from the producer biomass, culture medium, and reagents used during PHA isolation [21,22]. Therefore, most studies evaluate methods based on extraction yield, polymer purity, molecular weight, and the polydispersity index [23,24,25]. The reported yield and purity values reflect the metrics and experimental conditions used in the original publications and should be interpreted comparatively rather than as absolute performance indicators. Other factors may also be relevant, such as the consistency of the recovered material and the presence of chemical impurities, most notably immunostimulatory lipopolysaccharides typically found in Gram-negative bacteria [26].

In this review, we focus on the wide range of isolation and purification strategies that can be used to obtain PHAs from microbial biomass at a quality suitable for technological applications.

## 2. Physical Methods for PHA Isolation

### 2.1. Physical Disruption of Cellular Biomass

Several physical methods, including milling, HPH, ultrasonication, and gamma irradiation, were tested for disrupting cell biomass (Table S1). The application of physical methods for releasing PHAs from cells represents an effort to reduce or replace solvent use during the extraction process.

Ball-mill grinding has been used as a standalone method for isolating scl-PHAs (PHB, hydroxybutyrate-*co*-hydroxyvalerate, PHBV) from both native and dried biomass, resulting in the disruption of the cell walls of PHA-accumulating bacteria and subsequent release of PHAs. Reported PHA yields vary widely, ranging from 35 to 74%, but in all cases the purity of the recovered polymer remained low in the absence of additional biomass pretreatment [27,28,29,30]. Ball milling was used to obtain PHBV from dry *Cupriavidus necator* biomass and yielded a purity of 46%. When an additional pretreatment step was introduced, namely, solubilizing the biomass in a buffer containing 5% (*w*/*v*) sodium dodecyl sulfate (SDS), the isolation efficiency increased to 100%, and the polymer purity reached 94% [29].

The ball-mill approach was originally described by Tamer et al. [27,28] in combination with pretreatment of native *Alcaligenes latus* biomass, which was exposed to thermal shock prior to milling. Poorly selected pretreatment led to low isolation efficiency and required repeated grinding to disrupt the biomass effectively. PHB yields were determined gravimetrically after applying various physical and chemical pretreatments or their combinations, for example, SDS, NaOH, NaCl with heat, or combinations of salts, alkali, and heat. Selecting an appropriate pretreatment is crucial for optimizing the number of milling cycles. Alkaline pretreatment with NaOH proved to be the most effective method for isolating PHB from native *A. latus* biomass, although it required immediate neutralization to prevent PHB hydrolysis. This resulted not only in the desired breakdown of cell material but also in unwanted micronization of PHB.

Ball milling remains an attractive option for PHA recovery due to its low energy demand and its independence from biomass concentration, making it suitable even for low-PHA cultures. Its drawbacks include the relatively low purity of the obtained polymer and the need to optimize several parameters, particularly the choice of pretreatment for native or dried biomass, as well as the relatively long duration of the process [31].

Although ball milling is a commonly used method for bacterial biomass intended for PHA isolation due to its easy implementation at both laboratory and industrial scales, its intensive action, particularly on dried biomass, may damage PHA granules and thereby reduce the quality parameters of the isolated polymer. In contrast, HPH can be used to disrupt the biomass of PHA-producing strains, but it is less versatile than ball milling because it becomes inefficient at both very low and very high biomass concentrations [28]. This method has been tested effectively for isolating scl- (PHB or PHBV) and mcl-PHAs (poly(3-hydroxybutyrate-*co*-3-hydroxyhexanoate), P(3HB-co-3HHx)) from producing microorganisms in the range of 85–98% and purities of 43–98% (*A. latus*, *C. necator*, *Haloferax mediterranei*, *Methylobacterium* sp., and *Pseudomonas* sp.) [27,28,32,33,34]. Wet *A. latus* biomass was exposed to thermal shock followed by disruption using HPH, although this approach was limited by frequent clogging of the homogenizer [27]. HPH treatment of dry *Methylobacterium* sp. V49 biomass produced PHB with 80% purity and a 95% (*w*/*w*) yield. The main limitation of HPH was the low purity of the recovered polymer, caused by residual non-polymeric cell components. Purity increased to 95% only when HPH was combined with a pretreatment step, such as 5% (*w*/*v*) SDS [32]. Additionally, the high pressure applied during homogenization can cause PHA micronization, leading to polymer losses [28].

HPH can process samples of *C. necator* biomass with relatively high cell densities (20–100 g/L CDW), and polymer micronization can be avoided by limiting the process to no more than three cycles at 600–1200 bar, with higher pressure (approximately 1000 bar) recommended already in the first cycle [33]. However, in the case of PHBV extraction from *H. mediterranei*, a single cycle at 1000 bar was sufficient to achieve a high yield (85%) as well as high purity (90%). This may be related to the cell wall structure of *H. mediterranei* and the lack of peptidoglycan compared to the other tested producers, making it more susceptible to this PHA isolation approach. However, a negative effect of increasing pressure on molecular weight was observed, with a decrease as the pressure increased from 50 to 1000 bar [34].

Sonication generates rapid, extreme pressure cycles in a liquid, improving mass transfer and phase dispersion. It is particularly effective in viscous systems. Isolation of scl-(PHB) and mcl-PHAs (poly(3-hydroxyoctanoate); PHO) has been carried out on both native and dried biomass (*Bacillus* sp., *C. necator*, *H. mediterranei*, and mixed microbial culture (MMC)), but it showed low effectiveness in the range of 44.9–80% [29,30,35,36] The potential of sonication (20 kHz, 45 min) as a standalone isolation method was also tested on frozen and resuspended *H. mediterranei* biomass. Although PHBV granules were released and could be separated from cell debris by simple centrifugation, the study did not evaluate the overall extraction efficiency [35]. This limited effectiveness was confirmed for *C. necator*, where sonication of dry biomass yielded 47% PHBV with 40% purity [19], supporting previous observations that this organism is relatively resistant to ultrasonic disruption [37]. Sonication of wet *Bacillus* sp. biomass produced a 44.9% yield after 5 min of exposure [30]. When applied to native biomass from a mixed PHB-producing culture, ultrasound alone had limited impact on cell disruption, required high energy input, and was more effective only at low biomass concentrations (3 g/L), where approximately 14% cell lysis was observed [36]. Sonication appears to be more suitable as a pretreatment step before solvent extraction rather than as a standalone method for PHA isolation [36,38,39], or the pretreatment of the PHA-containing biomass is required prior to sonification [36].

Gamma irradiation was also tested as a physical cell-disruption method for PHA isolation by exposing native *Bacillus flexus* biomass to doses of 5–40 kGy [40]. After irradiation, the biomass was suspended in chloroform and homogenized. Without this step, PHB and/or the HB/HHx homopolymer appeared in the chloroform phase. Gamma irradiation increased the yield from 18 to 45% (*w*/*w*), raised the polymer’s molecular weight from 1.5 × 10^5^ to 1.9 × 10^5^ Da at 10 kGy, and slightly improved tensile strength (from 18 to 20 MPa). Applying gamma irradiation to the biomass of the PHA producer is advantageous because relatively low radiation doses provide effective cell disruption, improving polymer extractability and enhancing its material properties. Despite these benefits, the method is associated with extremely high capital costs and the risk of generating hazardous waste, which restricts its use on an industrial scale [41].

Based on the available results, the physical methods can be considered moderately effective, but they are not universally applicable to all PHA-producing microorganisms, and their efficiency depends on both the type of polymer produced and the producer. Their main advantage compared to other isolation techniques is the minimal damage to the polymer. They are also cost-effective and environmentally favorable, as they generally do not require chemical reagents. However, the drawbacks include long processing times, high energy demand depending on the physical method chosen, the need to optimize multiple parameters, and the difficulty of scaling these approaches for industrial production. Moreover, physical methods often need to be combined with solubilization techniques using organic solvents, surfactants, or chemical digestion [31].

### 2.2. Solvent Extraction

Another group of physical methods relies on dissolving PHAs in a suitable solvent. While traditional approaches use chlorinated solvents, newer methods focus on non-chlorinated alternatives (Table S1).

Before solvent-based extraction, unit operations are typically carried out to separate the PHA-containing biomass from the cultivation medium. This step increases the specific amount of PHAs per unit of processed material. Biomass is most often separated by centrifugation, filtration, or sedimentation. Extraction can then proceed either from native or dried biomass, and this choice plays an important role in selecting an appropriate solvent. Although dried biomass has the advantage of longer storage stability before extraction, native biomass can offer a less energy-intensive process if the release of PHA is sufficiently facilitated [42]. In addition to the use of solvents, it is common to subject the biomass to physical, physicochemical, or chemical pretreatments that help disrupt the cell structure while preserving the integrity of the polymer [14]. The following section focuses on the different pretreatment methods that have been applied prior to solvent-based PHA extraction.

#### 2.2.1. Pretreatments Prior to Solvent Extraction

##### Physical Pretreatment Methods

Thermal pretreatment is the most commonly used physical method applied to the biomass of a PHA producer before solvent extraction. In conventional extraction protocols employing chlorinated solvents, water removal is essential to ensure proper saturation of the biomass with the non-polar organic solvent. Thermal pretreatment also weakens cell structures by denaturing proteins and destabilizing the outer cell membrane. In the case of *C. necator* DSM 545, heat treatment was additionally shown to denature PHB depolymerase, the enzyme responsible for polymer degradation, which is located on the surface of P3HB granules [43]. The temperature range varies but is typically around 50–60 °C to prevent cell damage [23,44,45,46,47,48,49]. Higher temperatures, such as 90–150 °C, have also been reported [24,50,51,52], but these conditions reduce the molecular weight of the biopolymer. For example, a decrease in PHBV molecular weight from 1465.75 to 204.05 kDa was observed when drying was carried out at 120 °C [51].

Freezing of native biomass (*Bacillus cereus*, *C. necator*, *Pseudomonas putida*, and MMC) prior to analysis is another commonly used pretreatment method [51,53,54], and in some studies it is followed by lyophilization [48,55,56,57,58,59]. From an operational perspective, thermal drying requires considerably lower costs than lyophilization, which is a more complex process both in terms of execution and equipment requirements. This is likely why lyophilization is not used as a pretreatment at an industrial scale [13,60].

Other pretreatment approaches include milling to increase the specific surface area of the extraction biomass and improve the efficiency of solvent extraction [61,62]. In addition to conventional ball and blade milling, several physical pretreatment methods were evaluated prior to PHBV extraction with an organic solvent, and although none of them resulted in complete cell disruption, the highest PHBV yields were obtained when the biomass was pretreated using HPH [62].

Ultrasound-based pretreatment is another widely used method to enhance solvent extraction of PHAs [24,48,62,63]. Its mode of action differs between native and dried biomass. In native biomass, ultrasound disrupts cell structures before extraction and releases PHA into the surrounding medium, allowing the polymer to dissolve passively in the organic solvent without the need for the solvent to cross diffusion barriers. For dried biomass, it facilitates faster solvent penetration into the material and accelerates the establishment of equilibrium between the extraction solvent and the polymer-containing matrix.

Ultrasound was applied to disrupt cells in native *A. latus* biomass before PHB extraction with chloroform, using 60 cycles of 30 s sonication followed by 5 s of cooling. However, the study did not assess how this pretreatment affected the extraction yield [63]. Sonication was also applied to native biomass of PHBV-producing microorganisms isolated from wastewater treatment plants, but the pretreatment proved relatively ineffective, yielding only 7% [62]. A combined chloroform-ultrasound approach was tested for isolating PHB from dried *C. necator* biomass, but the study did not compare the efficiency of this method with chloroform extraction alone [38]. Freeze-dried *C. necator* biomass was resuspended in water containing PHB and sonicated in ten 2 min cycles with glass beads, but this had no positive effect on the extraction efficiency when methylene chloride was used as the solvent [24]. Other studies likewise reported no beneficial effect of ultrasound during the pretreatment of dry biomass [42,48,64].

Although sonication combined with solvent extraction can be effective for mcl-PHAs, the application of sonication during extraction with an acetone:heptane mixture (1:1; *v*/*v*) was observed to be more efficient for mcl-PHA recovery from dried *P. putida* biomass at 37 kHz than at 80 kHz during a 10 min treatment [65]. The lower frequency likely produced a stronger cavitation bubble collapse. The highest mcl-PHA content was 37.1 ± 2.2%, and sonication did not significantly affect the thermal properties of the biopolymer.

Prior to solvent extraction, supercritical CO_2_ extraction was applied as a pretreatment of native MMC biomass, with temperature and pressure being key parameters of this process. The use of methanol in supercritical CO_2_ extraction resulted in increased PHB purity (81%) with a yield of 83%. The molecular weight of PHB was not reduced during this process, in contrast to the use of the dimethyl carbonate (DMC) method [66]. However, the applicability of this process remains questionable due to its high financial costs.

To improve the yield of PHA extraction with organic solvents, a pre-extraction step can be applied using a solvent that does not dissolve PHA but effectively removes lipids, which could otherwise reduce the purity of the final biopolymer. Moreover, mildly polar solvents can also remove residual moisture after drying. Acetone is the most commonly used for this purpose [32,67,68,69,70], but *n*-hexane [52,71], water, methanol, ethanol, and isopropanol have also been tested [44,64,72]. When lyophilized *C. necator* biomass was pretreated with acetone, the PHB yield increased to 70% with 96% purity, compared with 55% yield and 92% purity without pretreatment. The processing time was also reduced four-fold [67]. Several polar solvents, including water, methanol, ethanol, and isopropanol, were tested as pretreatment agents and compared with untreated dried *C. necator* biomass containing PHB. Ethanol performed best, yielding 84 ± 1.6% PHB compared with 39 ± 2.1% without pretreatment after 30 min of mixing at a 1:25 (*w*/*v*) ratio at room temperature. Extending the pretreatment to 2 h enabled 100% PHB recovery from *A. latus* biomass after subsequent chloroform extraction using a Soxhlet apparatus [64].

##### Chemical Pretreatment Methods

A common pretreatment for the extraction of PHA from bacterial biomass (*Bacillus* sp., *C. necator*, *H. campisalis*, *Methylobacterium* sp., and MMC) used before solvent extraction is chemical treatment with sodium hypochlorite (NaClO) [24,44,45,56,73,74,75,76,77,78,79,80]. Hypochlorite treatment is used almost exclusively together with chlorinated solvents, most often chloroform, either as an initial step before solvent extraction [79] or simultaneously. The simultaneous use of NaClO and chloroform is referred to as the dispersion method [44,45,56,73,74,75,78]. Methylene chloride has also been used as the chlorinated solvent in combination with NaClO for the isolation of PHB from *C. necator* [14]. The main drawback of the dispersion method is its negative impact on the properties of the biopolymer, particularly its molecular weight [56,73,74]. A 3% (*v*/*v*) NaClO solution used with chloroform reduced the molecular weight from 1.2 × 10^6^ to 1.0 × 10^6^ Da after 1 h at 30 °C, while yielding PHB of 97% purity [74]. Higher hypochlorite levels (30%; *v*/*v*) in a 1:1 (*v*/*v*) mixture of chloroform and aqueous NaClO produced a 91% PHB yield with 97% purity [73]. When applied to lyophilized *B. cereus* cells, a 1:1 (*v*/*v*) mixture of chloroform and 15% (*v*/*v*) NaClO at 38 °C for 1 h gave a 30% PHB yield with 95% purity, comparable to conventional chloroform extraction [56]. Interestingly, in this study, the use of NaClO did not significantly affect the PHB’s molecular weight (8.9 × 10^5^ Da), polydispersity (3.1), or melting temperature (171.71 °C), and the extraction time decreased from 12 to 1 h. It is worth noting that when chloroform extraction was carried out using a Soxhlet extractor, the resulting PHB exhibited a higher molecular weight (1.1 × 10^6^ Da) and a lower polydispersity index (1.75), whereas the melting temperature remained comparable (169.71 °C).

Pretreatment of PHA-producing biomass (*C. necator* and MMC) with NaClO has also been tested in combination with non-halogenated solvents such as dimethyl carbonate (DMC), ethanol, and acetone. However, these studies did not evaluate the impact of this approach on the properties of the recovered polymer [24,76,77,80], and the NaClO pretreatment was applied to native biomass [76,77].

These results suggest that hypochlorite pretreatment can be a suitable approach for dry biomass when followed by extraction with an appropriate solvent, typically a chlorinated one. During the dispersion method, NaClO degrades cellular material, while the hydrophobic PHA granules released from the cells dissolve directly in chloroform, preventing their further degradation by NaClO. It is essential to carefully optimize the NaClO concentration and the pretreatment duration, as inappropriate settings can lead to moderate or even substantial reductions in PHA molecular weight.

Non-ionic surfactants can also be used as pretreatment before solvent extraction, mainly to improve yields in mixed microbial cultures. Tween 20, Brij L4, and Triton X-100 have been tested on freeze-dried MMC biomass prior to extraction with DMC [81]. Tween 20 proved to be the most effective, giving a yield of 53 ± 2% and a polymer purity of 93.9%. Its presence increased the amount of recovered PHAs by 50% compared with DMC extraction without pretreatment.

Alkaline solutions such as NaOH are also used for the pretreatment of PHA-containing biomass. When 0.1 N NaOH was applied to lyophilized *P. putida* biomass with mcl-PHAs, it removed most cellular material but also caused polymer losses because the viscous suspension was difficult to centrifuge. A 2 h treatment yielded a PHA purity of 44%. When this pretreatment was followed by extraction with a non-halogenated solvent (acetone), the purity increased significantly, and the molecular weight of the mcl-PHAs was not markedly affected [72].

In summary, pretreatment is essential for efficient PHA extraction, but the optimal approach is highly system-dependent. Physical pretreatments can improve solvent accessibility but often require careful parameter control to avoid polymer degradation or yield loss. Chemical pretreatments, particularly those based on NaClO, are highly efficient in removing cellular material, yet they present a substantial risk of reducing the molecular weight of the polymer if the concentration or exposure time is not strictly optimized. Surfactants and mild alkaline solutions can enhance extraction yields in specific cases, although their performance remains strongly dependent on biomass composition and solvent choice. Each method enhances recovery under certain conditions while posing distinct risks to polymer integrity.

#### 2.2.2. PHA Isolation Using Halogenated Solvents

PHA extraction works through a two-step action of the solvent: (i) it first increases the permeability of the cell wall, allowing the polymer to escape, and (ii) then dissolves and removes the polymer from the biomass. For this purpose, halogenated solvents are used most often, particularly chlorinated ones. The use of halogenated solvents for PHA isolation was first described by Lemoigne [82], who extracted the polymer using chloroform. Later, Baptist [83] tested methylene chloride as well as a methylene chloride:ethanol mixture for isolating P3HB from the biomass of *Bacillus megaterium* and *Rhodospirillum rubrum*.

Chlorinated hydrocarbons, especially chloroform, are the most widely used solvents for PHA extraction. Chloroform is mainly applied to dry biomass of PHA producers (Table S1), typically at room temperature to 100 for 2–48 h and at ratios of 1:10–1:100 (*w*/*v*). The method is highly efficient, giving 87–96% PHB recovery with 93–98% purity [42,55,56,67,84,85]. Extraction at 4 °C (36 h; 1:60; *w*/*v*) has also been tested, although its impact was not evaluated [58]. Chloroform extraction of PHAs from native biomass of *C. necator*, *B. flexus*, or MMC can be used only when combined with a pretreatment step, most commonly NaClO dispersion [77,86,87] or gamma irradiation [40]. Chloroform extraction is considered one of the most effective methods for isolating PHAs and is often regarded as the reference method. This technique enables the recovery of biopolymers with high purity (93–98%) and high molecular weight (above 1.0 MDa), although the polydispersity index shows a wide range of values (1.3 to 3.2) [53,55,56,58,67].

Methylene chloride is used less often (laboratory temperature (LT)-55 °C, 1:10–1:300 (*w*/*v*), 0.75–5 h), yielding 25–98% scl- or mcl-PHAs (*B. megaterium*, *C. necator*, *A. latus*, *P. putida*, and MMC) with 78–95% purity (Table S1). The use of 1,2-dichloroethane, 1,1,2-trichloroethane, or 1,1,2,2-tetrachloroethane has also been reported for *B. megaterium*, *P. putida*, or *C. necator*, although with lower efficiency compared to chloroform or methylene chloride [53,57,67,83].

After extraction, PHAs from *P. putida*, *B. cereus*, *C. necator*, *H. alkalicola*, or MMC are recovered from the primary solvent either by evaporation or by precipitation using a secondary solvent (anti-solvent), which reduces the solubility of PHAs in the primary extraction medium. This step helps decrease the amount of impurities present in the extract, particularly lipids and proteins from both scl- (PHB or PHBV) and mcl-PHAs (poly(3-hydroxyoctanoate); PHO). Anti-solvents such as methanol or ethanol, typically chilled and applied at ratios of 1:4 to 1:10 (*v*/*v*) for 0.5–2 h, are most used; mixtures such as acetone/ethanol (1:1, *v*/*v*) have also been tested. Hexane has likewise been used as an anti-solvent at ratios of 1:1 or 1:2 (*v*/*v*). Anti-solvent treatment also reduces endotoxin content, which is strictly regulated for materials intended for medical applications, although endotoxin levels are already very low when chlorinated solvents are used [23,29,42,53,56,57,58,77,78,84,87].

This method has several drawbacks. It requires very large quantities of solvents, often 20- to 100-fold the biomass mass containing PHAs. Under laboratory conditions, this limitation can be partially mitigated by using a Soxhlet extractor, which enables the use of smaller solvent volumes in a continuous cycle [24,50,56,64,72,88]. Soxhlet extraction also operates at milder temperatures, reducing the risk of polymer degradation. In contrast, high-temperature chloroform extraction can disrupt the morphology of P3HB granules from *C. necator* and decrease molecular weight due to chain scission, which negatively affects polymer properties [67]. Such structural changes limit the applicability of the polymer. Although its high purity still allows medical use, the altered physical properties hinder applications such as fiber production [14]. Another issue is the high viscosity of extracts when the P3HB content exceeds 5% (*w*/*v*), which increases interactions with cell wall residues and makes their removal more difficult [31,89]. A major disadvantage is the environmental and health burden associated with chlorinated solvents. These are toxic, poorly biodegradable, and require costly waste-management procedures. These factors substantially increase operational and capital costs, limiting the feasibility of this method for large-scale production.

#### 2.2.3. PHA Isolation Using Non-Halogenated Solvents

Efforts to limit the negative impact of halogenated solvents on the environment and living organisms have prompted the exploration of non-halogenated solvents for PHA extraction, including alkanes, alcohols, ketones, esters, ethers, amides, and sulfoxides [42,48,76,77,86] (Table S1). For the development of a process applicable on an industrial scale, extraction procedures employing non-halogenated agents require optimization of parameters that influence the overall course and outcome of extraction. A key parameter in selecting the most suitable extraction agent is the determination of PHA solubility in various organic solvents [86]. Compared with halogenated solvents, non-halogenated solvents offer advantages such as lower toxicity and reduced environmental burden. However, their industrial application remains limited by low process yields and the financial costs associated with the high energy demands of these processes.

##### Alkanes

Among alkanes, *n*-hexane is frequently reported in the literature as an extraction solvent. It has been applied for both scl- (*C. necator* and *A. latus*) and mcl-PHAs (*P. putida*), although with differing levels of efficiency. PHB from *C. necator* with a purity of 89% was obtained using *n*-hexane extraction, although the overall yield was low at only 2.6% (*w*/*w*) when compared with chloroform-based extraction [77]. Extraction of PHB from dried *C. necator* biomass with a Soxhlet extractor resulted in a yield of 49 ± 3.2%, corresponding to a 1.7–1.8-fold lower yield than that achieved with chlorinated solvents (chloroform, methylene chloride) [64]. *N*-hexane was evaluated as an extraction solvent for isolating mcl-PHAs (PHO) produced by *P. putida* GPo1 over 24 h at LT, using a solvent-to-biomass ratio of 1:15 (*w*/*v*), resulting in a 53% yield with 93% purity. When extraction was performed at an elevated temperature (50 °C), yields approached 76% with a purity of approximately 90%. However, methylene chloride and acetone provided more favorable yields (86 and 80%, respectively) [57].

A notable advantage of using *n*-hexane is the lower endotoxin content in the recovered polymer. Extraction of PHAs (PHO) from *P. putida* with *n*-hexane or tetrahydrofuran (THF) resulted in lower endotoxin levels than extraction with methylene chloride or ethyl acetate [57]. Higher extraction temperatures caused a marked increase in endotoxin content. These endotoxins could be removed by precipitating the polymer through cooling the extract to 0–5 °C, yielding a product with >97% purity (*w*/*w*) and endotoxin levels in the range of 10–15 EU/g PHO.

Based on these results, *n*-hexane does not appear to be a suitable extraction solvent for the isolation of either scl- or mcl-PHAs.

##### Alcohols

Another option for extraction is the use of alcohol-based solvent systems. In the literature, methanol, ethanol, and propanol have most frequently been tested for the isolation of scl-PHAs from *C. necator* (PHB) [76,77,86]. Polymers obtained with these solvents exhibited high purity (97–99%) and molecular weights comparable to those of polymers isolated by reference chloroform extraction. The main drawback of alcohol-based systems is their lower yield relative to chloroform extraction. Yields were generally lowest when propanol was used (23–28.5%), whereas methanol and ethanol produced higher yields (52.1–81.2%) [48,76,77,86].

These extraction protocols were tested with both native and dried PHB-producing biomass (*C. necator* and *P. putida*), with native biomass typically giving 1.3- to 1.8-fold higher yields [48,77]. For dried biomass, pretreatment with a hypotonic buffer containing lysozyme followed by drying was used. Effective application of alcohol solvents generally also requires pretreatment of native biomass of *C. necator* or *B. cereus* with NaClO (4–10% Cl_2_), which disrupts cellular structures and improves polymer accessibility [76,77,78]. Extraction itself is then carried out with polar solvents at elevated temperatures (50–100 °C) for a relatively short duration (0.5–1 h), yielding between 60.0 and 92.3%. The final product is isolated by precipitation using *n*-hexane in a 1:1 (*v*/*v*) ratio or by cold ethanol in the same ratio [76,77].

Alcohol-based solvents can extract PHAs with high purity and preserved molecular weight. However, these benefits are offset by lower extraction yields, the need for biomass pretreatment, and the higher energy demand caused by elevated extraction temperatures. As a result, alcohol-based extraction methods are limited by their operational complexity and lower overall efficiency.

##### Ketones

Another group of non-halogenated solvents tested for PHA extraction includes ketones, most commonly acetone, cyclohexanone, methyl isobutyl ketone (MIBK), and methyl ethyl ketone (MEK) [54,69,70,72,76,90,91,92,93,94]. These solvents have been applied to both native and dried biomass containing either scl- or mcl-PHAs (Table S1).

Acetone was used for scl- (PHB or PHBV) and mcl-PHAs (PHO, poly([R]-3-hydroxy-ω-undecenoate-co-3-hydroxy-ω-nonenoate-co-3-hydroxy-ω-heptenoate; PHUE) extraction from different producers (*C. necator*, *P. putida*, *H. mediterranei*, *Burkholderia sacchari*, and *Rhodovulum sulfidophilum*, MMC). Using acetone to isolate PHB from native *C. necator* biomass at 50 °C yielded a polymer of high purity (98%), but with low extraction efficiency (82.6%) compared with chloroform extraction (96.0%) [76]. As in other non-halogenated extraction systems, pretreatment of biomass with 10% (*v*/*v*) NaClO was required. Temperatures up to the boiling point of acetone have also been applied for isolating scl- and mcl-PHAs from dried biomass of *C. necator*, *H. mediterranei*, or *P. putida*. In scl-PHAs (PHB or PHBV), yields remained low (1–3%), but increasing the extraction temperature to 130 °C raised the yield to 16.9% [42]. A dramatic improvement in scl-PHA extraction efficiency from dried biomass was observed under conditions of 7 bar and 120 °C for 20 min, resulting in a 91.6% yield and 98.4% purity without affecting molecular weight or thermo-analytical properties [69]. For mcl-PHAs (PHO), yields between 72.2 and 99.0% were achieved at extraction ratios of 1:15 (*w*/*v*) over 1–24 h [53,57,72]. Finalization of the product was carried out by rotary vacuum evaporation, by cooling-induced precipitation, or by precipitation with methanol [42,53,57,69,72] or pentane [94]. Despite the high purity of the recovered polymers, acetone extraction is limited by low yields and additional disadvantages, including the formation of volatile organic compounds and the risk of explosive mixtures when high-pressure conditions are used. But acetone represents an attractive option for PHA extraction due to its high extraction efficiency, the possibility of using water as an anti-solvent, and the simplification of downstream processing through direct solvent evaporation without significant polymer degradation [94].

Cyclohexanone has also been identified as a promising solvent for isolating scl-PHAs such as PHB and PHBV from native (recombinant *Escherichia coli* and *C. necator*) and dried biomass (MMC, *B. sacchari*, and *R. sulfidophilum*) [70,92,93,95]. Extraction of PHB from native biomass of recombinant *E. coli* using cyclohexanone for 5 min at 90 °C resulted in an 80% PHB yield [90]. Extraction of PHB from dried *B. sacchari* biomass at 120–130 °C and a ratio of 1:67 (*w*/*v*) produced a 93% yield and 98% purity [92]. Increasing the extraction temperature to 120 °C allowed PHB yields of up to 99% from dried *C. necator* biomass within 3 min. Subsequent precipitation with methanol (1:10; *v*/*v*) resulted in 99% purity [70]. PHBV was isolated from freeze-dried *R. sulfidophilum* biomass by cyclohexanone within 10 min at 125 °C with a yield of 98% [93]. PHBV extraction from MMC using cyclohexanone at 130 °C for 3 h was effective [95].

In all cases, the molecular weight and polydispersity index of the polymers (PHB and PHBV) were comparable to those of material obtained via chloroform extraction or commercial standards. A major limitation of cyclohexanone lies in its industrial synthesis route from benzene, which is not considered environmentally sustainable. Practical applicability of cyclohexanone is also limited by a strong odor even under fume hood conditions, safety concerns related to the hot filtration step, and the need for additional purification to obtain acceptable polymer purity [95].

The use of MIBK and MEK for isolating scl- (PHBV) and mcl-PHAs (P(HB-co-20 mol% HHx)) has also been investigated [54,94]. MIBK was able to extract 92% of PHBV from MMC biomass with a purity of 96% at 140 °C for 1 h, but extraction using DMC or acetone was more effective [94]. MIBK demonstrated greater suitability than MEK in terms of extraction efficiency and mcl-PHA quality. Extraction at 120 °C for 1 h at a 1:10 (*w*/*v*) ratio was effective for both native and dried *C. necator* biomass. Among non-halogenated solvents tested for mcl-PHA extraction from native *C. necator* biomass, MIBK showed the highest potential, reaching an 84% yield and 99% purity. A drawback of this method was the fractionation of the extracted mcl-PHAs according to HHx content, although full polymer precipitation could be achieved using anti-solvents such as *n*-hexane or *n*-heptane at ratios of at least 1:3 (*v*/*v*) for 1 h at LT.

In summary, ketone-based extraction systems represent a versatile group of non-halogenated solvents capable of isolating both scl- and mcl-PHAs with high purity. Their performance varies widely and depends on the solvent used, biomass type, and extraction conditions. However, their practical application is limited by low yields under standard conditions, the need for biomass pretreatment, the formation of volatile and potentially explosive mixtures, and sustainability concerns related to solvent production.

##### Esters

Non-halogenated ester solvents applied in PHA extraction include the cyclic ester γ-butyrolactone, the alkyl carbonates DMC, 1,2-propylene carbonate (PC), and ethylene carbonate (EC), as well as organic esters such as ethyl acetate and butyl acetate.

The applicability of γ-butyrolactone as an extraction solvent for dried *C. necator* biomass is limited, as it yielded only 45% (*w*/*w*) PHB even at elevated temperatures [70].

Linear and cyclic alkyl carbonates are considered one of the most environmentally favorable alternatives to halogenated solvents. Extraction of PHAs from native or dried biomass using DMC or EC is considered highly effective, providing high extraction yields, high polymer purity, and preserved molecular weight. When applied to dried biomass of MMC or *P. putida*, containing either PHB or PHBV, yields of 71.88% with purity above 91.2% were obtained within 1.0–4 h at 60–140 °C and ratios of 1:20–1:40 (*w*/*v*), with precipitation achieved using *n*-hexane or ethanol at 1:1 or 1:4 (*v*/*v*) [48,51,66,94,95,96,97,98]. A notable advantage of alkyl carbonates is the ability to extract PHA directly from native biomass of *C. necator* or MMC obtained after fermentation [51,77,96,99]. Native *C. necator* biomass pretreated with 10% (*v*/*v*) NaClO was extracted with EC (150 °C; 1 h) and subsequently precipitated with ethanol (1:1; *v*/*v*), yielding 98.6% PHB with a purity above 98% [77]. Direct extraction of native *C. necator* biomass using DMC resulted in PHB yields of 73 ± 8% at 90 °C for 120 min [99]. Extraction of PHBV from MMC using DMC produced yields of 66 ± 8% with purity above 99% [51].

Extraction by PC from dried *C. necator* biomass at 55–130 °C and a ratio of 1:10 (*w*/*v*) produced low yields of 33–42% and purity levels of 82–83% [29,42]. Higher efficiency was achieved when PC was applied to native *C. necator* biomass, yielding 95% PHB at 130 °C for 30 min, followed by 48 h precipitation with acetone (1:2; *v*/*v*) [100].

Across studies using alkyl carbonates, PHB was obtained with high purity (84–98%) and molecular weights in the range of 7.4 × 10^5^ to 1.3 × 10^6^ Da, suitable for thermoplastic applications. Overall yields typically exceeded 95–98% (*w*/*w*), comparable to chloroform extraction. Moreover, PHAs isolated using EC and PC exhibited lower crystallinity and higher elasticity [48,77,100,101]. EC is also economically advantageous, costing roughly half as much as chloroform. The main drawbacks of using these solvents are their high energy demand and the need to precisely control the extraction temperature. Temperatures below 120 °C do not extract all intracellular PHAs, while higher temperatures increase the yield but also reduce the molecular weight of the biopolymer [100,101].

Organic esters have also been used with varying success, most commonly ethyl acetate, as well as butyl acetate and ethyl propionate. Ethyl acetate has been tested for the extraction of both scl- and mcl-PHAs from dried and native biomass (*Aeromonas hydrophila*, *A. latus*, *C. necator*, *P. putida*, MMC) at LT-125 °C for 1–24 h at ratios of 1:10–1:300 (*w*/*v*), producing yields of 29–99% for scl-PHAs and 72.2–80% for mcl-PHAs, with purity levels between 86 and 100% [48,53,61,64,86,88]. Use of butyl acetate resulted in low yields when extracting mcl-PHAs (P(HB-co-20 mol% HHx)) from dried *P. putida* biomass (33 ± 3% at 100 °C for 4 h at 1:500 (*w*/*v*)) [54], although extraction of PHO achieved 80% yield and 92% purity following precipitation with methanol (1:5; *v*/*v*) at −20 °C for 24 h at 1:15 (*w*/*v*) [57].

The advantages of organic esters lie in their ability to extract PHAs from native biomass with high yield and purity while preserving the polymer’s molecular weight. Their limitations are the high energy input required due to the elevated extraction temperatures and the need to optimize conditions for each producer strain and polymer type, given the considerable variability reported in the literature.

##### Ethers

Ethers have also been investigated as extraction solvents, including cyclic and aromatic ethers such as THF [57,80], 2-methyltetrahydrofuran (2-MTHF) [51,80], methyl tert-butyl ether (MTBE) [53], and anisole [92].

THF was evaluated as one of several solvents for extracting mcl-PHAs (PHO) from dried *P. putida* biomass, yielding 80% after 24 h at LT using a 1:15 (*w*/*v*) ratio, although the resulting polymer purity (84%) was lower than that achieved with other non-halogenated solvents [57]. A similar efficiency (78.3%) was also observed during PHA extraction from dried MMC biomass [80].

For scl-PHAs (PHBV) from MMC, biobased solvents such as 2-MTHF or cyrene were shown to be more effective than halogenated solvents, but only for PHBV polymers with lower molecular weight. 2-MTHF also performed well with native biomass, achieving a yield of 73 ± 1% after 1 h at 80 °C and a 1:10 (*w*/*v*) ratio, compared with 62 ± 3% from dried biomass, while product purity remained high in both cases (99%) [51]. Comparable results were reported for single-step extraction of PHBV from MMC with 2-MTHF, yielding 78.3 ± 11.9% polymer with 93.2 ± 2.4% purity. Thermal analysis confirmed that the material had properties similar to commercial PHBV [80]. Another advantage of 2-MTHF is its recyclability; about 96% of the solvent can be recovered after extraction with a purity comparable to commercial 2-MTHF. However, the number of regeneration cycles after which the solvent remains usable for PHA extraction still needs to be established.

MTBE has also been applied to extract mcl-PHAs ((poly([R]-3-hydroxyoctanoate-co-3-hydroxyhexanoate); PHOHH and PHUE) from dried *P. putida* biomass (80 °C; 4 h at a 1:15 (*w*/*v*) ratio) [53]. MTBE produced lower yields (49–57% for PHOHH; 50–55% for PHUE) compared with chloroform, which gave 88–90% for both polymers. Polymer purities remained high (95–98%) in both cases. The suitability of this solvent depends on the balance between environmental impact and toxicity, even though this comes with lower extraction efficiency. Limitations include the lower molecular weight of the recovered PHAs, reduced viscosity compared with halogenated solvents, and this method requires longer extraction time and higher temperature.

Anisole has been reported as a promising solvent for extracting scl-PHAs (PHB) from dried *B. sacchari* biomass, achieving a 97% yield and 98% purity at 120–130 °C using a 1:67 (*w*/*v*) ratio after precipitation with ethanol [92]. While effective over a short extraction time, this approach requires high temperature and pressure as well as specialized equipment capable of maintaining such conditions.

Overall, ethers (2-MTHF and anisole) represent promising alternatives for PHA extraction, especially when solvent recyclability and reduced environmental impact are priorities. Their efficiency has been demonstrated particularly for scl-PHA extraction within relatively short time frames at elevated temperatures, where they may outperform halogenated solvents. The choice of ether strongly depends on the PHA type, the form of biomass, the desired polymer properties, and the technical capabilities of the laboratory or production facility.

##### Other Solvents (Amides and Sulfoxides)

Several amides, such as dimethyl acetamide (DMA) [59] and dimethyl formamide (DMF) [64,77], as well as sulfoxides, such as dimethyl sulfoxide (DMSO) [77], have also been evaluated.

DMA combined with LiCl has been used to extract scl-PHAs (PHB) from *C. necator* [59]. After optimization, extraction from dried biomass at 110 °C for 3 h yielded 95.3% PHB with a purity above 99%. An advantage of DMA is its ability to be recycled at least five times with minimal loss of efficiency. The extracted PHB showed physicochemical properties comparable to commercial PHB, including molecular weight, thermal stability, and crystallinity.

DMF was tested for PHB extraction from dried *A. latus* biomass pretreated with ethanol [64]. Under extraction conditions of 1:300 (*w*/*v*) at 30 °C for 5 h, followed by precipitation with cold methanol, they obtained a 68% yield of scl-PHAs (PHB), which was comparable to extraction with chlorinated solvents. Higher efficiencies (84–87%) were achieved with chlorinated solvents in a Soxhlet extractor. However, another study did not confirm the effectiveness of DMF when extracting PHB from native *C. necator* biomass pretreated with NaClO, even at 150 °C; the maximum yield achieved was only 30.1 ± 2% [77].

Similarly, unsatisfactory results were reported for DMSO, with PHB yields from *C. necator* ranging from 20.6 to 60.6% across extraction temperatures of 50, 100, and 150 °C [77]. Although PHB extracted using DMSO showed thermal stability and molecular weight comparable to commercial PHB, the extraction efficiency remained significantly lower than that obtained with chlorinated solvents (96%) or with non-halogenated solvents such as EC (98.6%).

DMA is the only solvent in this group that demonstrates real extraction potential. It achieves high yields and purity, preserves polymer properties, and can be efficiently recycled. Other solvents provide inconsistent or low yields of PHAs.

In summary, halogenated solvents remain the most efficient option for PHA isolation in terms of yield and polymer quality, but their environmental and safety drawbacks limit their applicability at larger scales. Non-halogenated solvents offer a reduced environmental burden, with acetone and DMC emerging as promising alternatives to chloroform. However, their performance strongly depends on solvent type, biomass pretreatment, and extraction conditions, and it typically requires balancing efficiency, energy demand, and polymer properties. It is necessary to verify the applicability of this more environmentally friendly extraction approach for each producer and each type of PHA produced, followed by process optimization. Table 1 summarizes the advantages and disadvantages of using halogenated and non-halogenated solvents for PHA isolation.

## 3. Chemical Digestion

### 3.1. PHA Isolation Using Sodium Hypochlorite

NaClO is a strong oxidizing agent capable of degrading proteins, lipids, polysaccharides, and nucleic acids, which makes it potentially useful for chemically breaking down the cellular material surrounding PHA granules. Its application for PHA isolation has been described in a limited number of publications [16,36,102,103]. The major limitation of this method is the substantial degradation of the biopolymer, reflected in a marked decrease in molecular weight [16,47,73,74,102,103,104,105]. Interestingly, the extent of PHA degradation, reported mainly for scl-PHA (PHB), was shown to depend on the producing microorganism. A lower degree of PHB degradation was observed in Gram-positive strains compared with Gram-negative ones, indicating that the susceptibility of PHA to NaClO treatment is influenced by differences in cell-wall structure [56].

NaClO is therefore used mainly as a pretreatment step (see Chemical Pretreatment Methods), but several studies have also applied it directly for PHA isolation from *C. necator*, *P. putida*, *Bacillus australimaris*, or MMC reporting process yields of 70–100% and polymer purities of 74–99% [16,47,102,103,104,105,106,107]. This approach has been applied to both scl- (PHB and PHBV) and mcl-PHAs (PHO), although it appears more effective for scl-PHAs [16,47,102,103,104]. In contrast, the efficiency for mcl-PHAs from *P. putida* is low, yielding only small amounts of P3HO when 10% (*v*/*v*) NaClO is used at LT for 5–60 min [105].

The most critical parameters include the pH of the NaClO solution and its concentration. A pH of 10.0 caused the least degradation of PHB [102]. Alkaline conditions are generally preferred, but when pH is reduced to 5.0–6.5, hypochlorous acid (HClO) becomes predominant. Because HClO is a stronger oxidizing agent than NaClO, this shift significantly accelerates polymer degradation. To improve PHB purity, acidification of the NaClO solution has been attempted using HCl, thereby increasing the HClO:NaClO ratio. A higher HClO content increased the process yield from 73 to 97% (*w*/*w*) and raised purity from 74 to 83% when applied to MMC biomass [16].

NaClO concentrations used for scl-PHAs range from 5 to 30% (*v*/*v*), applied for 5–204 min at room temperature or at 37 °C [16,23,36,47,102,103,104,106]. For mcl-PHAs, only 10% (*v*/*v*) NaClO has been tested [105]. Parameters such as temperature or biomass concentration were not identified as major factors influencing extraction efficiency. NaClO-based isolation was also shown to be effective even at high biomass concentrations, with only minimal reductions in PHB yield [103].

This method has been applied almost exclusively to lyophilized biomass, where poor homogenization of the dry material led to a 1.4- to 2-fold reduction in PHB yield [103]. Several studies have also applied NaClO to native biomass [36,47,106]. When 9% (*v*/*v*) NaClO was used for 3.4 h at LT, PHBV yields from native biomass reached 90% with a polymer purity of 99%, without any detectable reduction in molecular weight or polydispersity [47]. It should be noted, however, that this comparison was made against PHBV isolated by Soxhlet extraction with chloroform rather than a commercial standard. In most other studies, NaClO treatment caused substantial reductions in polymer molecular weight, typically 50–70% lower than that of commercially available PHAs, although polydispersity index values often decreased as well [16,102,103,104,105]. As a result, NaClO-based isolation remains limited by the pronounced molecular-weight degradation of the polymer compared with material recovered using halogenated solvents.

### 3.2. PHA Isolation Using Ionic Liquids

Ionic liquids (ILs) are ionic compounds with melting points below the boiling point of water. Their ability to disrupt cellular membranes makes them promising candidates for releasing PHA from producer cells. Several ILs have been evaluated for this purpose, most commonly 1-ethyl-3-methylimidazolium (EMIM) acetate, (EMIM) dimethyl phosphate (DMP), (EMIM) diethyl phosphate (DEP), and (EMIM) methyl phosphate (MP). These have been applied to scl-PHAs (PHB and PHBV) contained in either dry (lyophilized) or native (wet) biomass (*Synechocystis* sp., *Halomonas hydrothermalis*, and *R. sulfidophilum*, *C. necator*). Extraction conditions varied in biomass-to-IL ratio (1:10–1:30, *w*/*v*), duration (4–24 h), and temperature (LT, 60 or 80 °C) [29,93,108,109]. Reported yields ranged from 33 to 98%, with product purity between 1 and 86%. Final purification typically involved precipitation with chilled methanol, usually at a 1:4 (*v*/*v*) ratio.

The main limitation in the use of ILs is their viscosity. Low yields of PHBV from *C. necator* obtained using (EMIM) acetate were attributed directly to this factor at a biomass-to-IL ratio of 1:10 (*w*/*v*) at 85 °C for 3 h [29], and similar issues were documented in other studies [93,108,109]. Efforts to mitigate viscosity have included the addition of an antisolvent (typically methanol) directly to the reaction mixture after extraction [93].

Among the ILs evaluated for PHA isolation, (EMIM) (DMP) appears the most suitable. When *Synechocystis* sp. was extracted at a ratio of 1:30 (*w*/*v*) at 60 °C for 24 h, a PHB yield of 98% was obtained [108]. The highest yields reached 60% PHB from either dry or native biomass of *H. hydrothermalis*, with a purity of 86% using (EMIM) (DMP) [109]. Higher yields were achieved only with (EMIM) (MP) under conditions of 1:10 (*w*/*v*) at LT for 24 h, with reported solubilization of 98% PHB [108]. However, this result did not involve extraction from producer biomass but an assessment of the ability of the IL to dissolve purified PHAs in the presence of cyanobacterial biomass. When tested on producers accumulating PHB (5 wt%), the purity of the recovered polymer was only 30% [108]. Purity can be improved by subsequent purification steps such as methanol precipitation or adsorption of organic impurities on activated carbon.

In addition to their high viscosity, a further limitation of ILs is the low purity of the recovered biopolymer, as these solvents dissolve a broad range of cellular components. Most impurities, unlike PHAs, dissolve in methanol used for precipitation. Despite these drawbacks, ILs offer a notable advantage, namely their reusability. A portion of the ILs could be recovered over several extraction cycles. The first cycle yielded 70% of the (EMIM) (DEP), followed by 60% in the second cycle, and subsequent analyses confirmed that its structure remained intact [109]. Similarly, (EMIM) (MP) could be reused across five cycles with 98–99% recovery, although this was tested using biomass with low PHA content [108]. Despite such benefits, IL-based PHA extraction remains relatively uncommon, largely due to the cost of ILs.

### 3.3. PHA Isolation Using Surfactants

Surfactants can also be used to isolate PHA from microbial biomass. Their mode of action is based on interactions with the phospholipid bilayer of the cell membrane, which they disrupt through partial or complete solubilization in an aqueous environment. This destabilizes the membrane and leads to cell disintegration. The released phospholipids and other cell components then form micelles with the surfactant, allowing the PHA granules to be released [14]. Detergents applied in PHA isolation include anionic surfactants such as SDS, linear alkylbenzene sulfonates (LAS-99, Trilon M), sodium dioctyl sulfosuccinate (AOT) or fatty acid salts (ammonium laurate, sodium palmitate); cationic surfactants such as palmitoylcarnitine or cetyltrimethylammonium bromide (CTAB); and nonionic surfactants such as Triton X-100, IGEPAL CA-630, Brij 58, or Tween 20 [25,28,29,39,44,68,75,96,110,111,112] (Table S1).

SDS is the most frequently used surfactant and has been applied to both scl- (PHB and PHBV) and mcl-PHA (PHBHHx) producers (recombinant *E. coli*, *Halomonas* sp., *C. necator*, *Pseudomonas* sp., MMC). Isolation has been performed using both dried (usually lyophilized) and native (wet) biomass. Experimental variables included biomass-to-surfactant ratios of 1:1 to 1:60 (*w*/*v*), SDS concentrations of 0.1–12%, and extraction times ranging from 0.5 to 24 h at temperatures from LT up to 90 °C. Reported PHA yields ranged from 60 to 95%, with polymer purity between 79 and 99% [24,29,39,44,68,75,110,111].

SDS concentration is not the dominant factor. The conditions of PHA extraction, especially temperature, play a larger role. Increasing the temperature by 20 °C improved PHA purity from *C. necator* by up to 20%, even though the SDS concentration (0.625%; *w*/*v*) remained the same in both cases [111]. SDS is also more effective when the biomass has a high PHA content. When biomass contained only 33–45% PHAs, polymer purity dropped significantly, and an additional purification step was required. The producer type is important, as biomass from halophilic microorganisms must be desalted before SDS-based extraction to prevent interference. This is illustrated by the low scl-PHA yield of 7.85% obtained from *H. campisalis* using 0.1% (*w*/*v*) SDS at 37 °C for 30 min [44].

Because SDS solubilizes cellular material only partially, the resulting polymer purity is often lower than desired. SDS is often combined with physical disruption methods, including sterilization [75], HPH [32], sonication [39,111], or ball milling [29]. These methods are typically applied to dried biomass (*C. necator*, *Methylobacterium* sp.) and help enhance the solubilization of cellular debris. However, the choice of physical method must consider energy and time requirements so that SDS-based extraction does not become unnecessarily demanding. Chemical methods can also be combined with SDS for both native and dried biomass (MMC and *C. necator*), such as NaOH [110] or NaClO [27,113]. These combinations bring additional drawbacks, especially a negative impact on polymer molecular weight. For NaClO, further disadvantages include high cost, environmental concerns related to toxic waste generation, and long extraction times [28,29].

Despite these limitations, SDS still offers several advantages, such as rapid isolation, a simple workflow, the possibility of adding SDS directly to the cultivation medium without pretreatment, and potential scalability to industrial PHA production. Moreover, SDS does not reduce PHA molecular weight or alter its thermal properties [29,111]. Large-scale application is hindered by the substantial amounts of surfactant required, which generate large volumes of wastewater and increase overall production costs. SDS is also relatively expensive and exhibits acute aquatic toxicity at concentrations as low as 3–4 mg/L [114]. SDS can be removed and regenerated at low cost. Treatment with 2 M KCl precipitates SDS as poorly soluble potassium dodecyl sulfate, after which the surfactant can then be regenerated by ultrafiltration in the presence of excess sodium ions [115].

Other fatty acid salts have been tested as potential agents for PHA isolation, including ammonium laurate and sodium palmitate. These compounds are referred to as switchable anionic surfactants (SASs), whose main advantage is their ability to be regenerated. SASs can be reversibly converted from a neutral, water-insoluble form into an anionic, water-soluble form simply by adjusting the pH. The pH shift can be achieved by adding or removing dissolved CO_2_ from the aqueous solution [116], and up to 98% surfactant recovery has been reported [96]. The extraction of scl-PHAs (PHB) from dried *C. necator* biomass using ammonium laurate (200 wt%) for 3 h at 90 °C with a 1:60 (*w*/*v*) ratio can yield more than 99% biopolymer with a purity above 90% [96]. These results were comparable to those obtained with SDS (3.3%; *w*/*v*). The combination of an SAS with an additional method further increased both yield and purity of the isolated polymer; in this case, ammonium laurate was used together with NaClO. The optimal conditions for extracting scl-PHAs (PHBV) from MMC involved pretreating dried biomass with 5% (*v*/*v*) NaClO (1:100, *w*/*v*; 85° for 1 h), followed by extraction with ammonium laurate (2:1, *w*/*w*; 75 °C for 3 h); a 74 ± 8% yield and nearly 100% polymer purity were achieved. However, a reduction in the molecular weight of scl-PHAs was observed, although their thermal properties remained largely unchanged [25].

The benefits of using SASs include their applicability to native (wet) biomass, their recyclability, and their lower cost compared with SDS. The method yields PHB with lower molecular weight and reduced thermal stability relative to solvent-based extraction. Moreover, this approach does not ensure the removal of endotoxins, which limits the potential use of the biopolymer in medical applications.

Among other anionic surfactants employed for PHA extraction were Trilon M [29], AOT [68], and linear alkylbenzene sulfonate (LAS) [111,112]. Trilon M provided a relatively high yield of scl-PHAs (PHBH) (71%) from dried recombinant *E. coli* biomass after six h of incubation at 90 °C, but the purity of the recovered biopolymer remained low (57%). Similarly, AOT enabled the recovery of 92.6% PHB from dried biomass, with a purity of 85.8% after 1 h at 30 °C. When the temperature was increased to 37 °C, the purity improved to 89.2% [68]. LAS-99 (5% *w*/*v*) was used to isolate PHBV from lyophilized *C. necator* cells. Under optimized conditions (pH 3.77; 60 °C for 3 h), followed by reducing the LAS-99 concentration to 1% (*w*/*v*) and adjusting the biomass-to-surfactant ratio to 1:0.5 (*w*/*v*), the process yielded 86% PHAs with 88% purity. LAS appears to be more effective than SDS for the extraction of mcl-PHAs from *Pseudomonas* strains. By combining HPH (two passes), LAS (5 wt%, 2 h at 60 °C), and hydrogen peroxide (2.4 wt%, 1 h at 60 °C), an mcl-PHA yield of 89% with a purity of 93% was achieved [112]. Compared with SDS, LAS offers the advantages of biodegradability, minimal ecological impact on organisms commonly present in the environment, and considerably lower cost [111]. Moreover, it is effective even at lower concentrations than SDS [112]. Key limitations of this method remain, as the surfactant cannot be reused and the recovered polymer has relatively low purity compared with solvent-based extraction. As a result, additional purification is required, increasing overall isolation costs.

Cationic surfactants (palmitoyl carnitine or CTAB) are used less frequently [68,117]. Among the various surfactants tested, CTAB produced comparatively lower yields of PHAs from recombinant *E. coli* (92.8–93.4%) as well as lower purity (84.1–89.0%). The least effective surfactants in the study were the non-ionic compounds Triton X-100 and Tween 20 [68]. Palmitoyl carnitine (1.0 mM, 1:1, *v*/*v*; 30 °C for 1 h) was more effective than lysozyme for *C. necator* or A. *latus*, but only the amount of released soluble proteins was evaluated, not PHA yield or purity [117].

The main advantages of the selected surfactants are the speed and simplicity of the isolation process and their suitability for direct application to native biomass, which gives them potential for industrial use. The drawbacks include the low polymer purity, which often requires a combined extraction approach, as well as concerns about the environmental acceptability, limited surfactant recyclability, and negative effects on PHA properties.

### 3.4. PHA Isolation Using Osmotic Pressure

The use of salts in PHA isolation is based on their ability to affect the integrity of the cell membrane through osmotic stress. Depending on the type of PHA-producing organism, cell disruption can be induced either by high salt concentrations or, in halophilic producers, by a sudden decrease in salt concentration.

In non-halophilic bacteria, exposure to elevated salt concentrations (NaCl, 2–10 g/L) induces hyperosmotic stress, leading to membrane destabilization and improved access to intracellular PHA granules. This approach is typically applied to native (wet) biomass (*C. necator*, *Bacillus* sp.) containing either scl- (PHB) or mcl-PHAs (P(3HB-co-3HHx)) at elevated temperatures (30–60 °C for 1 or 3 h), followed by cooling to 4 °C, resulting in a combined physical and chemical effect that may release intracellular granules [27,45,71,118]. However, this process serves only as a pretreatment step, and subsequent isolation of PHAs requires additional methods such as physical disruption using a ball mill [27], supercritical CO_2_ extraction [118], or alkaline hydrolysis with NaOH [71]. This pretreatment was less effective for isolating scl-PHAs (PHB) from native *C. necator* biomass [27,118], but it proved effective for wet biomass of *C. necator* producing mcl-PHAs (P(3HB-co-3HHx)), where pretreatment with NaCl (8 g/L, 30 °C for 3 h) increased the mcl-PHA content from 83.9 to 97.5% and polymer purity from 82.2 to 97.7%. This treatment disrupted the cell membrane, making the cell wall more susceptible to subsequent NaOH hydrolysis, without affecting the biopolymer’s molecular weight or thermal properties [71].

For halophilic PHA producers such as *Halomonas* and *Haloferax*, cell lysis can be efficiently induced by exposing the cells to hypotonic conditions. In this approach, PHA isolation from halophilic cells relies almost exclusively on the combined effect of osmotic cell lysis and solubilization of cellular components using SDS [115,119]. In low-salt environments, cells rupture due to high internal osmotic pressure, and SDS then solubilizes the released components, improving the purity of the insoluble PHAs. Released PHA granules can be separated by sedimentation, centrifugation, or filtration. PHA isolation from native biomass of halophiles (*H. mediterranei* or *Halomonas halophila)* producing scl-PHAs (PHB and PHBV) typically employs hypotonic solutions containing 0.1–1.0% (*w*/*v*) SDS, incubated at LT up to 90 °C for 2–24 h [115,120,121,122]. Additional isolation and purification steps were still needed because the recovered PHAs contained both lipophilic and hydrophilic impurities, and the procedure often had to be repeated [120] or supplemented with treatment using 3 or 30% (*v*/*v*) NaClO [121,122]. In contrast, simply increasing the temperature to 70 °C for 2 h in the presence of an aqueous SDS solution (0.5%; *w*/*v*) was sufficient to achieve a polymer purity of 96.7 ± 0.3%. This method offers potential applicability even under high-cell-density cultivation. A drawback is the reduced molecular weight of the biopolymer (1050.4 kDa) compared with the polymer recovered using the conventional chloroform method (1581.9 kDa) [115].

These strategies exploit microbial osmo-adaptive mechanisms to recover intracellular PHA granules efficiently and gently, often without the use of solvents. In non-halophilic producers, high-salt treatments are mainly effective for mcl-PHAs, whereas scl-PHA-producing biomass often requires further optimization, such as longer NaCl exposure. For halophilic producers, hypotonic solutions provide an effective route to high-purity PHA granules from moderately halophilic bacteria, although limitations related to SDS use remain.

### 3.5. PHA Isolation Using Alkaline Digestion

In addition to externally added fatty acids, a similar effect can be achieved through endogenous fatty acids naturally present in the biomass. Alkaline digestion with NaOH, KOH, or NH_4_OH exploits this principle. Strong inorganic bases saponify lipids, disrupt the cell membrane, and generate anionic surfactants that solubilize proteins and other cellular components in the aqueous phase while leaving PHA granules intact. Alkaline treatment has been applied to both scl- (PHB and PHBV) and mcl-PHAs (PHBHHx, PHO), using either native or dried biomass of PHA-producing microorganisms (recombinant *E. coli*, *C. necator*, *Comamonas* sp., *Pseudomonas acidovorans*, and MMC), achieving yields ranging from 45 to 91.3% and purities of 78 to 98.5% [23,36,68,71,111,118,123]. Alkaline solutions have been used both as a pretreatment step to enhance protein release prior to other extraction methods [23] and as a standalone procedure for PHA isolation from cells [36,68,71,111,118,123].

Suitable conditions typically involve moderate temperatures (4–37 °C) and exposure times of 1–5 h and alkali concentrations of 0.05–0.5 N [23,36,68,123]. Higher temperatures and longer treatments can increase purity but negatively affect polymer properties, particularly molecular weight [36,68]. After treatment, the alkaline solution must be removed and the recovered polymer stabilized, most often by repeated washing of the centrifuged pellet with water [68] or with a water:ethanol mixture [71,123]. When ethanol is used, residual base must first be removed to prevent sodium ethoxide formation, which would increase alkalinity and further reduce molecular weight and dispersity [71].

An advantage of this method is that it can be applied directly to native biomass without substantial losses in yield or purity [68]. It is most effective for microorganisms with thin cell walls, where only limited polymer degradation has been observed [123]. Alkaline treatment appears suitable for mcl-PHAs, for which no negative effect on molecular weight has been reported [71]. The main limitation is its impact on polymer properties, which depends on the microorganism and treatment conditions, and most strongly affects the molecular weight. Compared with chloroform extraction, alkaline treatment typically yields PHAs with molecular weights 1.3–2.9 times lower.

### 3.6. PHA Isolation Using Acids

In addition to alkaline agents, acids such as HCl, H_2_SO_4_, or CH_3_COOH have also been used for chemical digestion [23,68,77,124,125]. In these systems, an equilibrium can form between ester bond cleavage and reformation, since protons act as catalysts for both hydrolysis and esterification [126].

Acidic hydrolysis of the cell wall generally provides relatively high scl-PHA (PHB) yields (79–90%) from recombinant *E. coli* or *C. necator* with polymer purities of 92.3–95.6%. Among the acids evaluated, H_2_SO_4_ was effective under mild conditions (3.5%, *v*/*v*; 30–80 °C for 1–6 h) [23,68]. When higher temperatures (125 °C for several hours) were applied, it caused up to a 9.7-fold reduction in molecular weight [124]. High acid concentrations also appear problematic. The treatment with 6 M HCl at 110 °C for 22 h resulted in structural damage, especially in the amorphous polymer fraction, despite a relatively high yield (63.2%) [125]. The use of weak acids such as acetic acid produced lower PHB yields (36%), although polymer purity remained high (97%) [77].

Overall, a major limitation of acidic extraction, similar to alkaline extraction, is the reduction in PHA molecular weight [23,124]. Another limitation for industrial-scale applications lies in the corrosive nature of acids, especially at high concentrations, which increases safety risks and necessitates more robust and costly processing equipment.

## 4. Biological Digestion

Enzymatic digestion relies on the selective activity of enzymes to isolate PHAs from producer cells. The breakdown of the cell wall depends on selecting enzymes capable of cleaving peptide bonds within the peptidoglycan layer. This process can be combined with lysozyme treatment to degrade carbohydrate components or with thermal pretreatment (85–145 °C, 1–45 min). Thermal pretreatment facilitates cell disruption and can be further supported by surfactants that promote the decomposition of cellular components. Anionic surfactants such as SDS and ethylenediaminetetraacetic acid (EDTA) are preferred, as cationic surfactants tend to cause aggregation of cell debris through interactions with negatively charged lipopolysaccharides present in Gram-negative bacteria. SDS solubilizes hydrophobic and amphiphilic membrane components by forming micelles smaller than 10 nm, while EDTA chelates divalent cations (mainly Mg^2+^ and Ca^2+^), destabilizing the outer membrane and improving enzyme penetration [127].

Biological digestion has been effectively applied to both native and dried biomass of microorganisms producing scl- (PHB and PHBV) and mcl-PHAs (*C. necator*, *Pseudomonas* sp., *Burkholderia* sp., *Halomonas* sp., *Bacillus* sp., and MMC). Depending on the choice of enzymatic preparation, the use of lysozyme, or thermal pretreatment in combination with surfactants (SDS, EDTA), process yields ranged from 88 to 93.5%, and polymer purity ranged from 90.6 to 98% [127,128,129,130,131,132] (Table S1).

Multiple enzyme classes belonging to hydrolases have been studied for enzymatic hydrolysis, including proteases, glycosidases, and lipases [29,127,130,133,134,135]. These enzymes can cleave microbial proteins, glycoproteins, and glycolipids that form the structural matrix of the cell biomass. For practical enzymatic digestion, biomass should be separated from the cultivation medium, as applying enzymes directly to a medium with 1–8 g/L biomass requires about twenty times more enzyme due to dilution [127].

Proteases achieve the highest solubilization of non-PHA cell mass (NPCM), reaching 53–95%, whereas glycosidases or lipases typically achieve only about 26%. Proteases degrade peptidoglycan tetrapeptides and also other cellular proteins that make up around 55% of the total biomass [130]. A combination of glycosidases and proteases is more effective [129,131].

Alcalase (subtilisin A) is one of the most commonly used proteases for PHA isolation due to its broad substrate specificity and effective activity under alkaline conditions (pH 7.5–8.6) at moderate temperatures (40–60 °C). Its application typically results in process yields above 90–91%, with polymer purity around 92.6%. For biomass treatment, alcalase activity is applied at 0.3–2.4 AU per gram of biomass [128,129,131]. Its required enzyme activity can be reduced by extending the reaction time [127]. The reported efficiency of alcalase is based on a two-step protocol that includes thermal pretreatment of native biomass in an autoclave, followed by alcalase treatment (50–60 °C; pH 8.5 for 15–20 min) in the presence of SDS (0.08–0.15 g/g biomass) and a second step combining lysozyme (0.005–0.01 g/g biomass; 30 °C; pH 7.0 for 15 min) with EDTA (0.4 g/g biomass). The resulting process is therefore a combined effect of enzymatic digestion and detergent-assisted disruption. Alcalase itself has the dominant contribution (71.5%) to the overall PHA yield [129]. Further analyses demonstrated that the properties of the isolated PHAs were comparable to those of PHAs obtained by chloroform-based extraction, except for molecular weight, which was reduced by 17%. This reduction was attributed primarily to the use of EDTA rather than to enzymatic treatment alone [131].

Other proteases, including trypsin (0.1–5%, *w*/*w*; 37 °C; pH 8.0 for 12–24 h), chymotrypsin (1.0% *w*/*w*; 37 °C; pH 8.0 for 12 h), bromelain (4.8%, *w*/*w*; 50–55 °C; pH 4.75–7.0 for 1–24 h), or papain (0.5–4.7%, *w*/*w*; 40–70 °C; pH 6.0–7.0 for 1–12 h), as well as their combinations, have also been tested for PHA isolation from *C. necator* or MMC. These enzymes provided good polymer recovery, although yields varied widely depending on the protease used, ranging from 61.3 to 90.9% [128,130,135]. The combination of trypsin and hydrogen peroxide resulted in a PHA yield of 91.6% with a purity of 90.9% from dry MMC biomass. When biomass was pretreated using supercritical CO_2_ extraction, the yield increased to 97.3% and the purity to 98.1% [135]. Protease-containing enzyme extracts produced highly pure polymer (97%) from *B. flexus* but achieved a low yield of only 45.3 ± 1.5% [136].

Among glycosidases, lysozyme has been used either after endogenous production by a recombinantly engineered producer or through exogenous addition in the form of a technical enzyme preparation. The addition of lysozyme to wet *C. necator* biomass yielded 75% PHAs, although with low polymer purity (41%) [29]. Furthermore, a lysis module consisting of holin and endolysin derived from bacteriophage T4 was introduced into three strains of *Halomonas bluephagenesis*. The results showed that the self-lytic system was equally effective compared to the use of exogenously added endolysin [134].

Purified enzymes are useful for research purposes, but crude enzyme preparations are more economically feasible for PHA isolation. Pancreatin (2.0%, *w*/*w*; 50–70 °C; pH 8.0 for 8 h), a mixture of amylases, lipases, and proteases, released 90.3–93.5% PHAs from *C. necator* or *Burkholderia cepacia* with purities of 62.2–90.6%. Moreover, the polymer properties were comparable to those of commercially available PHAs, including glass transition temperature, melting point, decomposition temperature, and molecular weight, although enzymatic activity led to an increased crystallization temperature [130,132].

Enzymatic PHA isolation offers high reaction specificity, mild processing conditions, low energy demand, and minimal polymer degradation. Its wider industrial use is limited mainly by enzyme cost, variable purity, and the need for additional purification steps. For broader applicability, it is essential to identify an efficient crude enzyme preparation that would significantly reduce the cost of this environmentally friendly isolation approach.

An alternative could be the use of enzymes naturally produced by various organisms. The digestive enzymes of mealworms (*Tenebrio molitor*) were used to isolate scl-PHAs (PHB and PHBV) and mcl-PHAs (PHBHHx) from dried *C. necator* biomass [137,138]. Mealworms produce proteases, amylases, cellulases, and lipases. After collecting the frass pellets, high concentrations of residual proteins remained, but treatment with 1% (*w*/*v*) SDS at 50 °C for 10 h resulted in 100% polymer purity, comparable to PHA obtained via chloroform extraction, without changes in melting temperature, molecular weight, or polydispersity [137,138,139]. A purification step was required, involving washing with water and 0.1 M NaOH, which yielded PHBHHx with a purity of 92–94% [138]. However, the biological digestion of *C. necator* biomass containing scl-PHAs (PHB) was described as effective without pretreatment [139]. The frass pellets were washed with water, resulting in a high PHB isolation yield of 99.7%. In addition, feeding mealworms with *C. necator* biomass increased feed utilization by up to 2.5-fold compared with values reported by Ong et al. [138].

In addition to invertebrates, vertebrates have also been tested. Sprague Dawley rats were used to isolate scl-PHAs (PHB) from dried *C. necator* biomass [140]. The purity of the resulting polymer was 89%, and its properties were comparable to those of PHB obtained by chloroform extraction. When an additional purification step using 2% (*w*/*v*) SDS was included, purity increased to 97%.

These studies clearly demonstrate that organisms can digest the cellular biomass of the production strain, while PHA granules remain intact and pass through the digestive tract unchanged. The remaining limitations are the necessity of a purification step and the relatively long duration of this isolation process.

Chemical digestion methods are effective in removing cellular material, but they are often associated with a reduction in polymer molecular weight. In contrast, biological digestion approaches are generally milder and better preserve polymer properties, although their efficiency is typically lower and they are rarely suitable as standalone isolation methods (Table 2).

## 5. Conclusions

There is a continuous need to identify and implement environmentally acceptable alternatives to conventional plastics. PHAs represent one such option because they are readily degradable by naturally occurring microbiota. However, their high production cost remains a major limitation, and the choice of isolation method significantly contributes to this expense. The most efficient isolation methods still rely on halogenated solvents, which allow rapid processing at relatively manageable costs, partly due to the possibility of recovering and reusing the extraction solvent.

Despite ongoing research aimed at identifying suitable PHA producers that would lower production costs, primarily through reducing the price of feedstocks, PHA isolation remains a major cost factor, as it typically involves biomass drying followed by chloroform extraction. Therefore, a key challenge is to identify viable alternatives to conventional extraction using halogenated solvents. The literature also reports inconsistent results even for identical producers and processing strategies, highlighting the need for critical reviews that systematically summarize current knowledge on PHA isolation.

Combining multiple environmentally acceptable methods appears to be a promising approach. While physical and chemical methods remain standard, the application of biological strategies using crude enzyme extracts or entire organisms for NPCM degradation represents a progressive direction. As highlighted in this review, successful industrial implementation will depend on the availability of efficient and low-cost enzyme preparations, which requires both fundamental research to identify suitable enzyme producers and the development of scalable enzyme production systems.

## Figures and Tables

**Table 1:** Comparison of PHA isolation using halogenated and non-halogenated solvents.

Type of Solvent	Category	Advantages	Disadvantages
Halogenated solvents	Chloroform, 1,2-dichloroethane, 1,1,2-trichloroethane, 1,1,2,2-tetrachloroethane, methylene chloride	Very high extraction yield (typically 87–96%) High polymer purity (93–98%) Preservation of high molecular weight Reference method with good reproducibility	High toxicity and environmental burden Large solvent consumption High costs of waste treatment and disposal Limited applicability at the industrial scale
Non-halogenated solvents	Alkanes (*n*-hexane)	Lower endotoxin content in the recovered polymer	Very low extraction yield, particularly for scl-PHAs Lower efficiency compared to halogenated solvents Unsuitable as a standalone extraction method Limited number of available studies
	Alcohols (methanol, ethanol, propanol)	High polymer purity (scl-PHAs, 97–99%) Preservation of the molecular weight of scl-PHAs	Lower extraction yield compared to chloroform Require biomass (dry) pretreatment Higher energy demand due to elevated extraction temperatures
	Ketones (acetone, cyclohexanone, MIBK, MEK)	Capable of extracting both scl- and mcl-PHAs High purity of scl-PHAs (acetone, cyclohexanone) or mcl-PHAs (MIBK) High extraction yield under optimized conditions	Low efficiency under mild conditions Requirement for high temperatures and/or pressures Risk of forming volatile or explosive mixtures
	Esters (γ-butyrolactone, DMC, PC, EC, ethyl acetate, butyl acetate)	High extraction yield and polymer purity Applicable to native biomass Preservation of polymer properties Lower toxicity and reduced environmental burden Some esters (DMC, ethyl, or butyl acetate) are less expensive than chloroform	High energy demand (due to the requirement for elevated extraction temperatures) Sensitivity to extraction temperature (reduction in molecular weight at elevated temperatures) Need for process optimization for each producer
	Ethers (THF, 2-MTHF, MTBE, anisole)	High extraction efficiency (scl- and mcl-PHAs) High polymer purity Potential for solvent recyclability (2-MTHF, anisole)	Requirement for high temperatures and pressures Need for specialized technical equipment Strong dependence on PHA type
	Other solvents—amides, sulfoxides (DMA, DMF, DMSO)	DMA provides high extraction yield and purity Good solvent recyclability (DMA)	Inconsistent performance Often lower yields compared with the reference method Limited number of available studies

**Table 2:** Comparison of PHA isolation using chemical and biological digestions.

Digestion	Type	Advantages	Disadvantages
Chemical digestion	NaClO	Efficient degradation of non-PHA cellular material High polymer recovery and purity are reported in several systems Applicable to scl-PHAs and selected mcl-PHAs	Pronounced degradation of the polymer molecular weight Strong dependence on pH and reagent concentration Lower effectiveness for mcl-PHAs Oxidative nature and environmental burden
Chemical digestion—ILs	(EMIM) acetate, (EMIM) (DMP), (EMIM) (DEP), and (EMIM) (MP)	Effective disruption of cellular membranes High extraction efficiency under optimized conditions Possibility of solvent reuse	High viscosity Co-solubilization of cellular components resulting in low polymer purity Need for additional purification steps High cost
Chemical digestion—Surfactants (SDS/LAS/SAS)	Anionic (SDS), linear alkylbenzene sulfonates (LAS, Trilon M, AOT), cationic and nonionic surfactants (Triton X-100, IGEPAL CA-630, Brij 58, or Tween 20)	Simple and rapid workflow Suitable for direct application to native biomass Generally preserves molecular weight and thermal properties	Polymer purity often insufficient without additional treatment Large volumes of wastewater generated Environmental concerns, particularly for anionic surfactants Limited endotoxin removal
Chemical digestion—Osmotic pressure	Salts	Enables cell disruption in osmotically sensitive or halophilic microorganisms Mild conditions without aggressive chemicals	Limited applicability to specific producer types Usually insufficient as a standalone isolation method Often requires combination with surfactants or chemical digestion
Chemical digestion—Alkaline digestion	NaOH, KOH, NH_4_OH	Effective removal of cellular material through membrane disruption and saponification Can be applied directly to native biomass	Negative impact on polymer molecular weight Typically requires subsequent extraction or purification
Chemical digestion—Acid digestion	HCl, H_2_SO_4_, CH_3_COOH	Efficient degradation of residual biomass High polymer purity reported under controlled conditions	Significant reduction in molecular weight Structural damage to polymer at elevated temperature or acid concentration Corrosive conditions limit applicability
Biological digestion—Enzymes	Alcalase, lysozyme, neutrase, bromelain, corolase, protease, papain, pancreatin, trypsin	Mild reaction conditions High selectivity toward cellular components Preservation of polymer molecular weight and thermal properties	High cost of enzymes Low efficiency as a standalone method Often requires combination with additional treatments
Biological digestion—whole organisms	Mealworms, rats	Environmentally friendly approach without harsh chemicals Selective degradation of cellular material in some systems	Low process controllability Long processing times Limited scalability and reproducibility

## Data Availability

No new data were created or analyzed in this study. Data sharing is not applicable to this article.

## Supplementary Materials

The following supporting information can be downloaded at: https://www.mdpi.com/article/10.3390/life16020269/s1, Table S1: Comparison of different PHA isolation methods and characterization of the obtained biopolymers.

## Abbreviations

2-MTHF	2-methyltetrahydrofuran
AOT	Sodium dioctyl sulfosuccinate
CDW	Cell dry weight
CTAB	Cetyltrimethylammonium bromide
DEP	Diethyl phosphate
DMA	Dimethyl acetamide
DMC	Dimethyl carbonate
DMF	Dimethylformamide
DMP	Dimethyl phosphate
DMSO	Dimethyl sulfoxide
EC	Ethylene carbonate
EDTA	Ethylenediaminetetraacetic acid
EMIM	1-ethyl-3-methylimidazolium acetate
HB	Hydroxybutyrate
HBHV	Hydroxybutyrate-*co*-hydroxyvalerate
HPH	High-pressure homogenization
IL	Ionic liquid
LAS	Linear alkylbenzene sulfonate
LT	Laboratory temperature
MEK	Methyl ethyl ketone
MIBK	Methyl isobutyl ketone
MMC	Mixed microbial culture
MP	Methyl phosphate
MTBE	Methyl tert-butyl ether
NPCM	Non-PHA cell mass
P(3HB-co-3HHx)	Poly(3-hydroxybutyrate-*co*-3-hydroxyhexanoate)
P3HB	Poly(3-hydroxybutyrate)
P3HBHV	Poly(3-hydroxybutyrate-*co*-3-hydroxyvalerate)
PC	1,2-propylene carbonate
PHA	Polyhydroxyalakanoate
PHO	Poly(3-hydroxyoctanoate)
PHOHH	Poly([R]-3-hydroxyoctanoate-*co*-3-hydroxyhexanoate)
PHUE	Poly([R]-3-hydroxy-ω-undecenoate-*co*-3-hydroxy-ω-nonenoate-*co*-3-hydroxy-ω-heptenoate
SAS	Switchable anionic surfactants
SDS	Sodium dodecyl sulfate
THF	Tetrahydrofuran
